# High-Precision Rotation Axis Calibration of Line-Structured Light Measurement System Using a Stepped Cylinder

**DOI:** 10.3390/s26134275

**Published:** 2026-07-05

**Authors:** Yuehua Li, Ziqi Jia, Haiyong Chang, Jingbo Zhou, Tiejun Li

**Affiliations:** School of Mechanical Engineering, Hebei University of Science and Technology, Shijiazhuang 050018, China; yuehua.hrbin@163.com (Y.L.); jiaziqi2000@163.com (Z.J.); 15533323823@163.com (H.C.)

**Keywords:** line-structured light measurement system, rotation axis optimization, stepped cylinder, system calibration

## Abstract

Line-structured light sensors have the advantages of simple structure, low cost, and high precision. Integrating the sensor with a turntable is an important strategy to achieve complete results of complex surfaces. To enhance measurement accuracy, we proposed a rotation axis calibration method using a stepped cylinder. Coefficients of the laser plane are firstly optimized based on the principle of cross-ratio invariance to ensure the sensor’s own accuracy. At each rotational angle, the laser plane intersects the cylinder, and corner points on the edges are extracted as feature points. Subsequently, an objective function is established by minimizing the average distance between rotated feature points and ideal circles and is solved using sequential quadratic programming method. After optimization, the average diameter error of the stepped cylinder is reduced by more than 90%. The relative errors of other typical features (ball diameter, cylinder diameter, groove depth) are all less than 0.15%. Measurement results of complex objects with different materials are also successfully obtained with their small features clearly observable.

## 1. Introduction

Line-structured light sensors (LSLSs) have the advantages of simple structure, low cost, and high precision [1], making them widely applied in three-dimensional (3D) measurements [2,3], industrial inspections [4,5], and character recognition [6]. LSLS, based on the laser triangulation principle, primarily consists of a laser line projector and a camera. During the measuring process, a laser plane is emitted from the projector and intersects the measured object. The intersection profile can be calculated using the central pixel coordinates of laser stripe, the equation of laser plane, and the camera’s intrinsic parameters [7,8,9].

Camera intrinsic calibration is highly mature. The classical one is Zhang’s method features high accuracy and easy to use [10]. There are also many methods for laser plane calibration like the three-point collinearity method [11], the planar mirror method [12], the concentric circle method [13], etc. Since LSLS can only capture two-dimensional (2D) profiles, it must be integrated with a linear stage or a turntable to achieve 3D results [14]. For linear scanning, only the surfaces facing the sensor can be captured [15]. Measurement range is also limited by the translation of the stage. Conversely, in a rotary scanning system, the object is placed on a turntable. The sensor can capture the geometrical information from different viewpoints, ensuring a more complete result with a more compact system setup [16]. Integrating LSLS with a turntable provides substantial benefits for 3D measurement, particularly for closed surfaces [17]. In such systems, the calibration of rotation axis is crucial for ensuring measurement accuracy.

Planar targets are widely utilized for rotation axis calibration. Chen et al. [18] determined the orientation of rotation axis by fitting the rotated corner points of a checkerboard target. To ensure a 360 degree view of the target, the camera was positioned above the axis. Liu et al. [19] enhanced the calibration accuracy by incorporating the target’s orientation information. Li et al. [20] replaced conventional linear scanning with rotary scanning and further developed a calibration and registration method for low-overlap fields of view. Hou et al. [21] unified the camera’s optical center at different angles and performed circular fitting to determine the turntable origin. Liu et al. [22] corrected the eccentricity error between the starting point of LSLS and the rotation axis, thereby achieving a comprehensive measurement result. Furthermore, Wang et al. [23] proposed a single-view method for rapid radial error measurement of eccentric shafts using line-structured light.

In the calibration of the rotation axis using a checkerboard target, it is a common practice to determine the circle center by fitting the corner points at different rotational angles. However, the coordinates of these corner points, derived via monocular vision, often suffer from limited accuracy. Moreover, as part of the LSLS, the camera is mounted laterally relative to the rotation axis. Consequently, the complete target is visible only within a restricted range of rotation angles. When these points are utilized for circle fitting, the center coordinates may have large deviations, thereby inducing significant calibration errors.

The utilization of dual-camera configurations has been proposed to improve calibration accuracy [24,25]. In such systems, an auxiliary camera is incorporated to capture the image of the checkerboard target from a more favorable view-point. The rotation axis is calculated within the auxiliary camera coordinate system using the aforementioned corner fitting method. Since the relative position between the two cameras has been calibrated, the rotation axis can be transformed into the measurement camera coordinate system. The introduction of the auxiliary camera enables the target’s rotational image to be observed from a more advantageous perspective, resulting in more accurate outcomes. Nevertheless, this approach increases system complexity, and the calibration precision is sensitive to the accuracy of the relative pose estimation between the cameras.

High-precision standard parts can also be employed for rotation axis calibration. Zong et al. [26] determined the spatial relationship between the rotation center and the camera by measuring a standard ball at various rotated angles. Ye et al. [27] placed a plane on the turntable and scanned it from multiple perspectives. The rotation axis vector was subsequently derived by analyzing the intersection points of the reconstructed planes. Zhu et al. [28] acquired point clouds of a rectangular workpiece before and after rotation and calculated the transformation matrix via point cloud registration. However, these methods are developed for fringe projection systems. For these systems, a relatively comprehensive 3D measurement result can be obtained for a specific rotation angle [29] and can be further enhanced using machine intelligence [30]. The feature localization errors caused by noise, blur, distortion, and imperfect calibration targets can also propagate through the geometric model and limit reconstruction accuracy [31]. In the case of the rotary scanning system with LSLS, only a single profile can be obtained for a given rotation angle. Guo et al. [32] aligned a cylinder with the turntable’s rotation axis. The cylinder axis was determined by fitting the centers of the intersection profiles and then regarded as the rotation axis. Nevertheless, the alignment process is intricate, and the alignment error directly influences the final result.

Here, a complete rotation axis calibration method is proposed based on a stepped cylinder. Stripe corner points located on the cylinder edges are firstly extracted as feature points. Subsequently, an objective function is established based on the average distance between the rotated feature points and the ideal cylinder edges. The optimization parameters are the coordinates of two points on the rotation axis. Our method does not require fine adjustment between the standard part and the rotation axis, ensuring high accuracy and ease of use.

## 2. Measurement Principle

**Figure 1:**
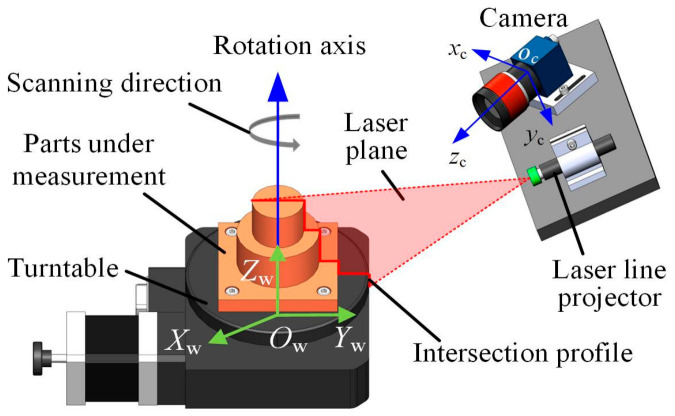
Measurement principle of rotary scanning system with LSLS.

Figure 1 illustrates the measurement principle of the rotary scanning system with LSLS. This system is primarily composed of a turntable, a camera, and a laser line projector. The laser line projector emits a laser plane that intersects the surface of the tested workpiece. The intersection profile can be calculated according to the pixel coordinates of stripe center points, the camera’s intrinsic parameters, and the equation of the laser plane. As the turntable rotates, current profile and the corresponding rotation angle are captured synchronously. Since the profile is computed within the camera coordinate system *o*_c_*x*_c_*y*_c_*z*_c_, the rotation axis within *o*_c_*x*_c_*y*_c_*z*_c_ must be determined to obtain complete 3D results. Subsequently, all the measurement points are transformed into the world coordinate system *O*_w_*X*_w_*Y*_w_*Z*_w_. For convenient data processing, the *O*_w_*Z*_w_ axis is aligned with the rotation axis, and the *O*_w_*X*_w_*Y*_w_ plane is set on the turntable.

**Figure 2:**
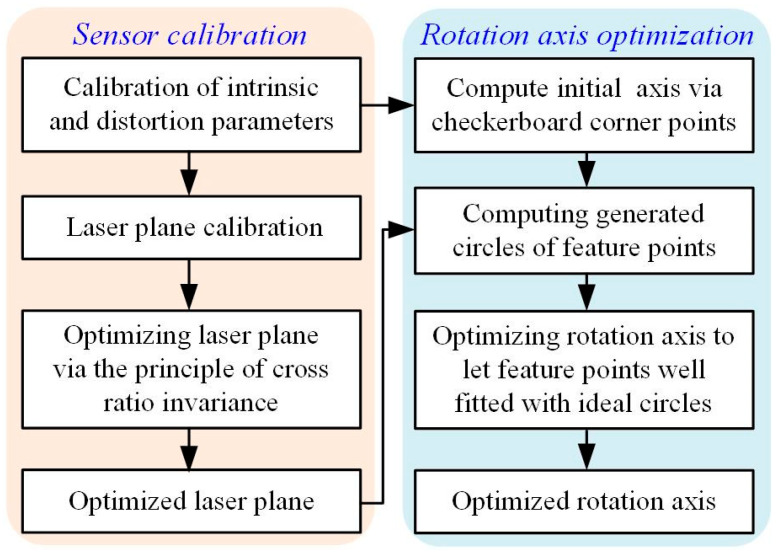
Procedures of sensor calibration and rotation axis optimization.

System calibration procedures are presented in Figure 2. To ensure the measurement accuracy of the LSLS, a laser plane optimization method is proposed based on the principle of cross-ratio invariance (CRI). The initial rotation axis is obtained using a checkerboard target and then refined with a stepped cylinder. When the laser plane intersects the cylinder, line segments are extracted to form a series of feature circles. A more precise rotation axis can be obtained by aligning these circles with the ideal ones. The complete calibration procedure will be elaborated in subsequent sections.

## 3. Calibration of LSLS

### 3.1. Measurement Model

Let **P**_c_(*X*_c_,*Y*_c_,*Z*_c_,1) represent the homogeneous coordinate of a point in *o*_c_*x*_c_*y*_c_*z*_c_, and let **p**(*u*,*v*,1) denote its corresponding pixel coordinate. According to the camera projection model, their relationship can be expressed as:

(1) $$s\cdot p=\left[\begin{array}{ccc} f_{x} & 0 & u_{0}\\ 0 & f_{y} & v_{0}\\ 0 & 0 & 1 \end{array}\right]P_{c}=K_{c}P_{c},$$

where *s* is the scale factor which represents the depth-related scaling between image coordinates and camera coordinates, **K**_c_ is the camera’s intrinsic matrix. *f_x_* and *f_y_* are the focal lengths, and (*u*_0_, *v*_0_) are the pixel coordinates of principle point.

Due to lens distortion, a deviation exists between the actual pixel point **p***_d_*(*u_d_*,*v_d_*) and the ideal pixel point **p**(*u*,*v*). The distorted pixel coordinates are given as:

(2) $$\left\{\begin{array}{c} u_{d}=u\left(1+k_{1}r^{2}+k_{2}r^{4}\right)+\left[2\rho_{1}uv+\rho_{2}(2u^{2}+r^{2})\right]\\ v_{d}=v(1+k_{1}r^{2}+k_{2}r^{4})+\left[2\rho_{2}uv+\rho_{1}(2v^{2}+r^{2})\right] \end{array}\right.,$$

where *r*^2^ = *x*^2^ + *y*^2^, *k*_1_ and *k*_2_ are radial distortion coefficients, *ρ*_1_ and *ρ*_2_ are tangential distortion coefficients. If **P**_c_ lies on the laser plane, its coordinates also satisfy the equation of laser plane:

(3) $$aX_{c}+bY_{c}+cZ_{c}+d=0,$$

where *a*, *b*, *c*, and *d* are the coefficients. For any point on the stripe center, its pixel coordinates after distortion correction can be solved according to Equation (2). For the camera intrinsic parameters and laser plane are known, the camera coordinates of this point can be obtained by solving Equations (1) and (3).

### 3.2. Laser Plane Optimization

**Figure 3:**
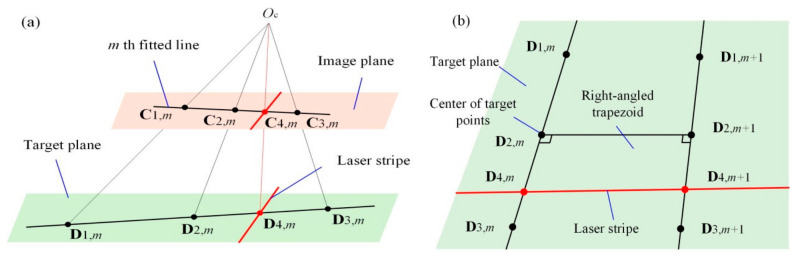
Interval distance computation for laser plane optimization. (**a**) Compute the value of |**D**_2,*m*_**D**_4,*m*_| via CRI, (**b**) compute the ideal interval distance within the right-angled trapezoid.

The laser plane equation is achieved by fitting a set of laser stripes, wherein its accuracy depends on the internal and external parameters of the camera [7]. To further enhance the sensor accuracy, we propose a laser plane optimization method based on the principle of CRI, as shown in Figure 3. In Figure 3a, **D**_1,*m*_, **D**_2,*m,*_ and **D**_3,*m*_ denote the central points of target dots in the *m*th row, while **C**_1,*m*_, **C**_2,*m*_ and **C**_3,*m*_ are their corresponding pixel points. **D**_4,*m*_ represents the intersection point between the laser stripe and the fitted line of the target centers, and **C**_4,*m*_ is its pixel point. The cross-ratio value for these points on the image plane can be expressed as:

(4) $$\lambda_{m}=CR(C_{1,m},\;C_{2,m};\;C_{3,m},\;C_{4,m})=\frac{C_{1,m}C_{3,m}/C_{2,m}C_{3,m}}{C_{1,m}C_{4,m}/C_{2,m}C_{4,m}},$$

Based on the principle of CRI, *λ_m_* can also be expressed by the interval distance as:

(5) $$\lambda_{m}=\frac{\left|D_{1,m}D_{3,m}\right|/\left|D_{2,m}D_{3,m}\right|}{\left|D_{1,m}D_{4,m}\right|/\left|D_{2,m}D_{4,m}\right|}=\frac{2\left|D_{2,m}D_{4,m}\right|}{I_{d}+\left|D_{2,m}D_{4,m}\right|},$$

where I*_d_* denotes the ideal interval distance of target points, and |**D**_2,*m*_**D**_4,*m*_| can be obtained by solving Equation (5), |**D**_2,*m*+1_**D**_4,*m*+1_| can be obtained in a similar manner. As shown in Figure 3b, **D**_2,*m*_, **D**_4,*m*_, **D**_2,*m*+1_ and **D**_4,*m*+1_ form a right-angled trapezoid. Therefore, |**D**_4,*m*_**D**_4,*m*+1_| for the *q*th placement of the target can be expressed by:

(6) $$I_{m,q}^{R}=\sqrt{{I_{d}}^{2}+{\left(\left|D_{2,m}D_{4,m}\right|-\left|D_{2,m+1}D_{4,m+1}\right|\right)}^{2}},$$

Since the pixel coordinates of **C**_4,*m*_ and **C**_4,*m*+1_ are known, the camera coordinates of their corresponding points **D**_4,*m*_ and **D**_4,*m*+1_ can also be calculated using the camera projection model and the laser plane equation in Section 3.1. The interval distance |**D**_4,*m*_**D**_4,*m*+1_| for the *q*th placement of the target is closely related to the coefficients of the laser plane, and can be denoted as $I_{m,q}^{W}$. When the position of the dot target is adjusted, multiple sets of distances between adjacent points are obtained. Using the coefficients of the laser plane as the optimization parameters, an objective function is established to minimize the root mean square value of the interval errors, as follows:

(7) $$E_{rms}^{I}={\left\{\frac{1}{Q(M-1)}\sum_{q=1}^{Q}\sum_{m=1}^{M-1}{\left[I_{m,q}^{W}(a,b,c,d)-I_{m,q}^{R}\right]}^{2}\right\}}^{\frac{1}{2}},$$

where *Q* is number of target placements, *M* is the rows of dots on the target. Subsequently, the sequential quadratic programming (SQP) method is employed to solve the optimization model [33].

## 4. Calibration of Rotation Axis

### 4.1. Calculate Initial Rotation Axis

**Figure 4:**
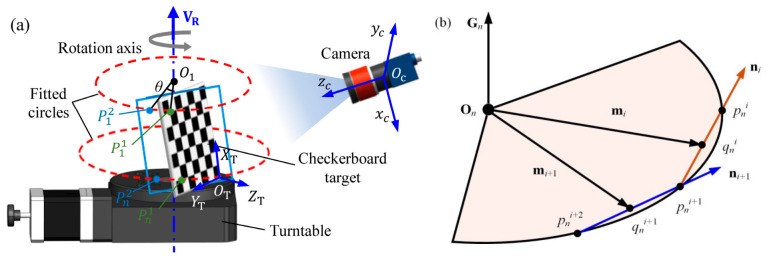
Computation of initial rotation axis. (**a**) Rotation axis computation with a checkerboard target, (**b**) circle center computation with corner points of the target.

A checkerboard target is employed to estimate the initial rotation axis, as shown in Figure 4a. The camera coordinates of the corner points are obtained using Zhang’s method. The normal vectors **G***_n_* for the *n*th circle is determined by plane fitting of the same corner point at different rotation angles. Geometrically, the perpendicular bisector of the chord connecting any two points on a circle passes through its center, as shown in Figure 4b. **O***_n_* is the center, $p_{n}^{i}$ and $p_{n}^{i+1}$ are the two adjacent points on the *n*th circle, $q_{n}^{i}$ is the midpoint, **n***_i_* = $p_{n}^{i}$ − $p_{n}^{i+1}$ is the vector of adjacent points, **m***_i_* = $q_{n}^{i}$ − **O***_n_* is the vector from **O***_n_* to $q_{n}^{i}$.

Based on the properties of a circle, it follows that **n***_i_* × **m***_i_* = 0. By substituting the expressions for **n***_i_* and **m***_i_*, we obtain **n***_i_***O***_n_ = **n**_i_*$q_{n}^{i}$. Furthermore, by integrating all 3D coordinate points derived from various rotation angles on the circle, the following equation can be derived:

(8) $$N_{n}O_{n}=L_{n},$$

where **N***_n_ =* [***n***_1_, ***n***_2_, *…*, ***n****_i_*]^T^, **L***_n_ =* [***n***_1_$q_{n}^{1}$, ***n***_2_$q_{n}^{2}$, *…*, ***n****_i_*$q_{n}^{i}$]^T^. Equation (8) represents an overdetermined system. By incorporating the constraint of the plane where the circle is located, we can obtain:

(9) $$\left[\begin{array}{c} (N_{n}{)}^{T}N_{n}\\ (G_{n}{)}^{T} \end{array}\right]O_{n}=\left[\begin{array}{c} (N_{n}{)}^{T}L_{n}\\ 1 \end{array}\right],$$

Let **Q***_n_* = [(**N***_n_*)^T^**N***_n_*, (**G***_n_*)^T^]^T^, **H***_n_* = [(**N***_n_*)^T^ **L***_n_*, 1]^T^, the coordinates of **O***_n_* can be expressed as:

(10) $$O_{n}={\left({\left(Q_{n}\right)}^{T}Q_{n}\right)}^{-1}Q_{n}^{T}H_{n},$$

The equation of the initial rotation axis can be achieved by linear fitting of these center points.

### 4.2. Rotation Axis Optimization Based on a Stepped Cylinder

**Figure 5:**
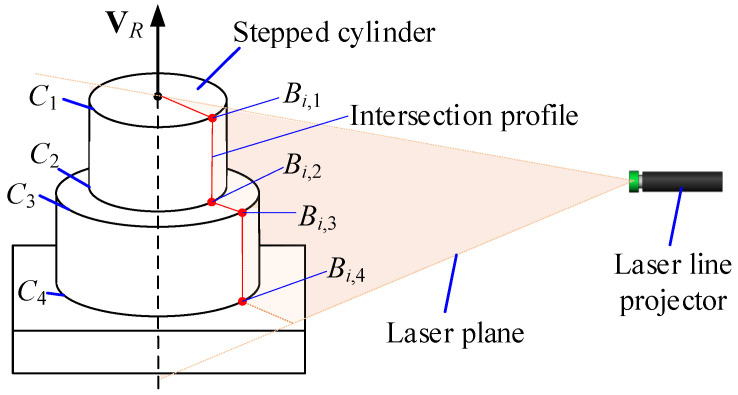
Illustration of feature points extraction.

As illustrated in Figure 5, the stepped cylinder consists of two cylinders and four circular edges, labeled as *C*_1_, *C*_2_, *C*_3_, and *C*_4_. These edges serve as ideal reference circles for rotation axis optimization. When the laser plane intersects the cylinder, a stripe with multiple line segments can be generated. The center points of each stripe segment are extracted using the improved gray gravity method [34] and then fitted via the Random Sample Consensus (RANSAC) algorithm [35]. The intersection points of adjacent fitting lines are designated as feature points, named *B_n_*_,1_, *B_n_*_,2_, *B_n_*_,3_ and *B_n_*_,4_ for the *n*th rotation. The camera coordinates of these points can be acquired using the sensor model discussed in Section 3.1.

**Figure 6:**
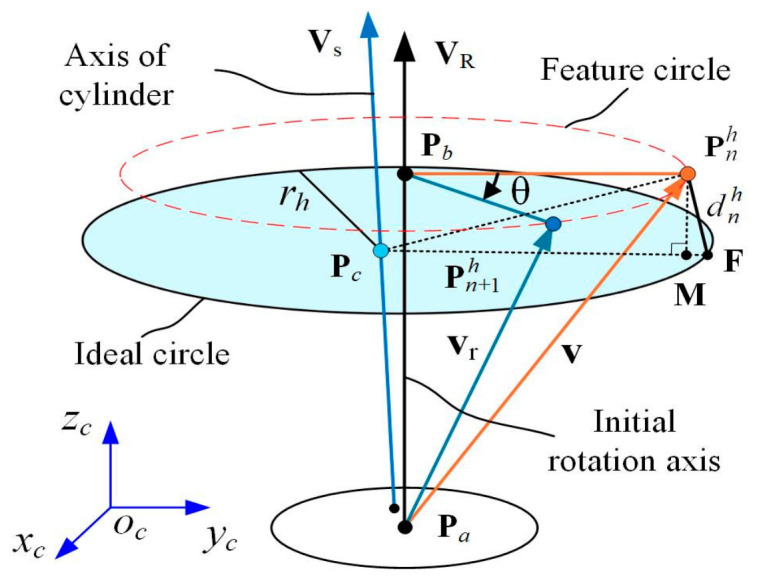
Illustration of rotation axis optimization.

The initial rotation axis can be determined by two distinct points, named **P***_a_* and **P***_b_*. The direction vector of the axis is expressed by **V***_R_* = **P***_b_* − **P***_a_*. Let **v** = $P_{n}^{h}$ − **P***_a_*, where $P_{n}^{h}$ are the 3D coordinates of a feature point after the *n*th rotation, and *h* denotes the index of the feature circles (*h* = 1, 2, 3, 4). When the feature points are well fitted with the ideal circles, the true rotation axis can be found, as shown in Figure 6.

Assuming **v**_r_ is vector **v** rotated by *θ* about **V**_R_, it can be expressed via Rodriguez’s rotation formula as:

(11) $$v_{r}=v\cos\theta +\left(\frac{V_{R}\times v}{\left|V_{R}\right|}\right)\sin\theta +v\left(\frac{V_{R}\cdot v}{{\left|V_{R}\right|}^{2}}\right)\left(1-\cos\theta \right),$$

The coordinates of a measured feature point $P_{n+1}^{h}$, after being rotated by *θ*, can be calculated as $P_{n+1}^{h}$ = **v**_r_ − **P***_a_*. For one complete rotation, the 3D coordinates of the feature points are collected and subsequently classified into four groups, with each group corresponding to a feature circle. The rotation axis of the measured points is determined by line fitting of the circle centers. This axis associated with measured points is then aligned with the cylinder axis. After that, the average distance between the feature points and ideal circles is computed for fine matching.

Assume **P***_c_* is the center of the *h*th ideal circle with the radius of *r_h_*, **V**_S_ the axis of the cylinder, **M** is the projection point of $P_{n}^{h}$ onto the plane of the ideal circle, **F** is the intersection point between **P***_c_***M** and the circle. According to the orthogonal projection principle, $\vec{P_{c}M}$ can be expressed by:

(12) $$\vec{P_{c}M}=\vec{P_{n}^{h}P_{c}}-\frac{\vec{P_{n}^{h}P_{c}}\cdot V_{S}}{{\left|V_{S}\right|}^{2}}V_{S},$$

The distance from $P_{n}^{h}$ to the ideal circle can be calculated by:

(13) $$d_{n}^{h}=\left|P_{n}^{h}-\left(P_{c}+r_{h}\frac{\vec{P_{c}M}}{\left|\vec{P_{c}M}\right|}\right)\right|,$$

Since the initial rotation axis can be determined by two points on it, named **P***_a_* and **P***_b_*. Their coordinate variations (Δ*x_a_*, Δ*y_a_*, Δ*z_a_*) and (Δ*x*_b_, Δ*y*_b_, Δ*z*_b_) can be taken as the optimization parameters. The average distance, $E_{avr}^{d}$, between the feature points and the ideal circles is taken as the objective value for fine matching, that is:

(14) $$E_{avr}^{d}=\frac{1}{HN}\sum_{h=1}^{H}\sum_{n=1}^{N}d_{n}^{h}\left(\Delta x_{a},\Delta y_{a},\Delta z_{a},\Delta x_{b},\Delta y_{b},\Delta z_{b}\right),$$

where *N* is the number of feature points for each circle, *H* denotes the number of ideal circles.

## 5. System Calibration

### 5.1. System Setup

**Figure 7:**
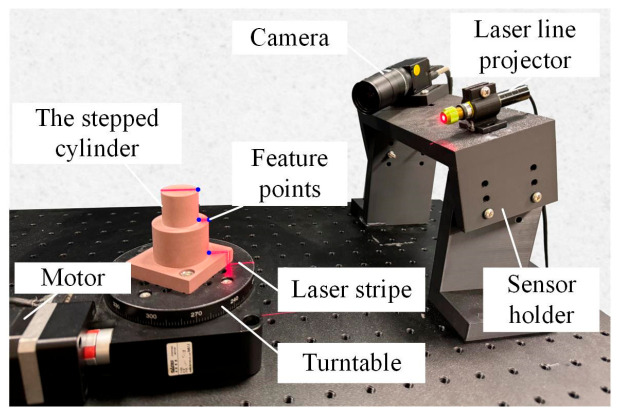
Measurement system.

Figure 7 illustrates the rotary measurement system. The laser line projector (Shengzuan Laser, Shantou, China) has a wavelength of 650 nm, a power of 5 mW and the minimum line width of 0.3 mm. The camera (MV-U300, MindVision, Shenzhen, China) has a resolution of 1280 × 960 pixels. The focal length of the lens can be manually adjusted from 4mm to 12 mm. Two targets are utilized for calibration. One is the Checkerboard target (CBC-80-G4.0-T1.0, PointVision, Shenzhen, China) with 17 × 19 square array. Each square has a side length of 4 mm with an accuracy of 1 μm. This target is used for camera calibration and computation of the initial rotation axis. The other one is a dot target (HAC-60-D3.0-T1.0, PointVision, Shenzhen, China) with 9 × 9 dot array. The interval distance between the dots is 6 mm, and the accuracy is also 1 μm. This target is used for laser plane optimization.

### 5.2. Camera Calibration and Laser Plane Optimization

**Figure 8:**
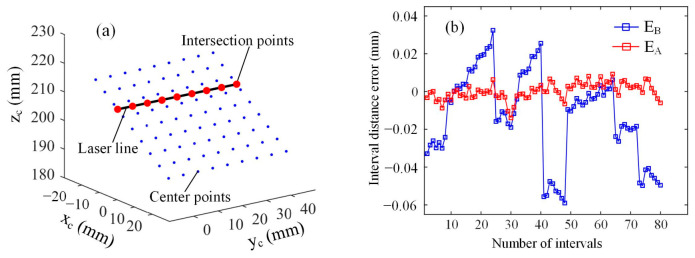
Laser plane computation and optimization. (**a**) Computed laser stripes in camera coordinate system, (**b**) interval errors before and after optimization.

The checkerboard target is placed at twenty distinct positions within the camera’s field of view to perform camera calibration using Zhang’s method [10]. The absolute mean reprojection error is 0.03 pixel, indicating high calibration accuracy. The laser plane is then calibrated by use of the dot target. When the laser plane intersects the target dot intervals, a laser stripe with uniform brightness can be ensured. Centerline of the stripe is extracted using the improved gray gravity method [34]. When the interval distance of the dots is known, the camera coordinates of the dot centers are calculated by use of Tsai’s method [36]. With the current plane equation of dot target, camera coordinates of the stripe center are computed using the method discussed in Section 3.1. The computed dot centers and the stripe line are shown in Figure 8a. By repositioning the dot target, multiple laser lines can be computed in the same way, and the laser plane equation is obtained by fitting these lines.

As detailed in Section 3.2, sensor accuracy is evaluated by comparing the measured interval distance $I_{m,q}^{W}$ with the reference interval distance $I_{m,q}^{R}$. In this experiment, the target is positioned at 10 distinct locations (*Q* = 10). For each location, eight interval distances are extracted (*M* = 8). For all the optimization using SQP, the tolerance is 10^−8^, and the maximum iterations are 150. The interval errors before and after optimization are denoted as E_B_ and E_A_, respectively. As shown in Figure 8b, the proposed laser plane optimization method can significantly reduce the interval error. The root mean square error (RMSE) decreased from 0.021 mm to 0.003 mm. This provides a robust foundation for subsequent measurements.

### 5.3. Initial Rotation Axis Calculation with Checkerboard Target

**Figure 9:**
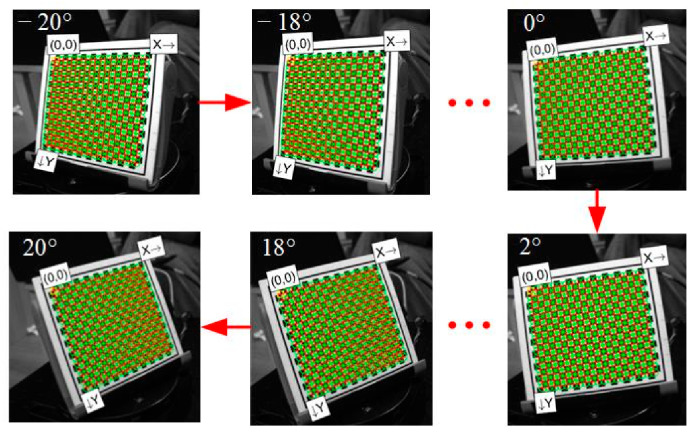
Target images with different rotation angles for axis calibration.

Detail procedures for rotation axis calibration using a checkerboard target are presented in Section 4. The target was placed on the turntable and rotated with an incremental step of 2°. Figure 9 illustrates the target images with extracted corners, whose camera coordinates are calculated using Zhang’s method [10].

**Figure 10:**
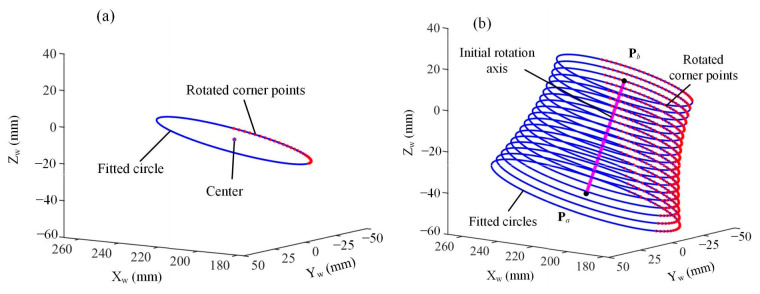
Method for computing initial rotation axis. (**a**) Fitting of a single circle, (**b**) computing initial axis with multiple circle centers.

As shown in Figure 10a, by fitting the same target corner at different rotation angles, the circle center can be determined using the method discussed in Section 4.1. For each target corner, the above-mentioned method yields one circle center. The rotation axis is achieved by fitting these circle centers, as illustrated in Figure 10b. For clear presentation, only the circles corresponding to one column of corner points are drawn. It is evident that clear target images can only be ensured within a limited rotation range of approximately 40°, one ninth of the circle. This results in a narrow range of the rotated points for circle fitting and significant errors are introduced. Consequently, substantial errors are introduced into the initial rotation axis, which has an adverse impact on the measurement accuracy of the scanning system.

### 5.4. Simulation Verification of Axis Optimization

**Figure 11:**
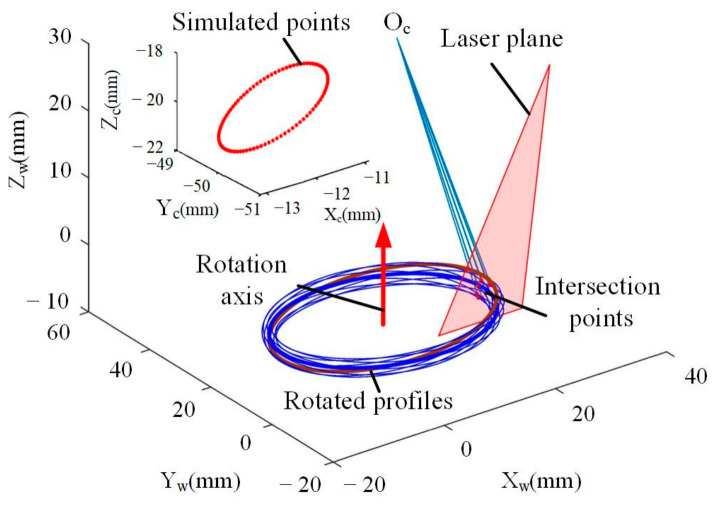
Illustration of measurement points generation in the simulation system.

To verify the feasibility of the proposed axis optimization method, a simulation analysis is also carried out. To simplify the analysis, the rotation axis is aligned with Z_w_ and one ideal circle C_1_ is placed within the simulation system. To replicate the misalignment between the cylindrical axis and the rotation axis, we apply rotation and translation transformations to C_1_. Subsequently, C_1_ is rotated around Z_w_. As shown by Figure 11, the intersection points between the laser plane and C_1_ at different rotation angles can be calculated as the simulated sampling points. Their coordinates are then transformed into the camera coordinate system to serve as the measurement points. Since the stepped cylinder has four ideal circles from C_1_ to C_4_, four sets of simulated points could be obtained using the same approach. Consequently, the axis optimization method can be verified by calculating the rotation axis based on these simulated points.

In practical measurement, the initial rotation axis calibrated using a checkerboard inevitably contains errors. For this reason, random deviations (Δ*a_x_*, Δ*a_y_*, Δ*a_z_*) and (Δ*b_x_*, Δ*b_y_*, Δ*b_z_*) are introduced to points **P***_a_* and **P***_b_*, respectively. The effectiveness of the proposed optimization algorithm can be verified by observing whether it can eliminate these deviations and achieve the ideal circles. The specific deviation values are shown in Table 1.

**Figure 12:**
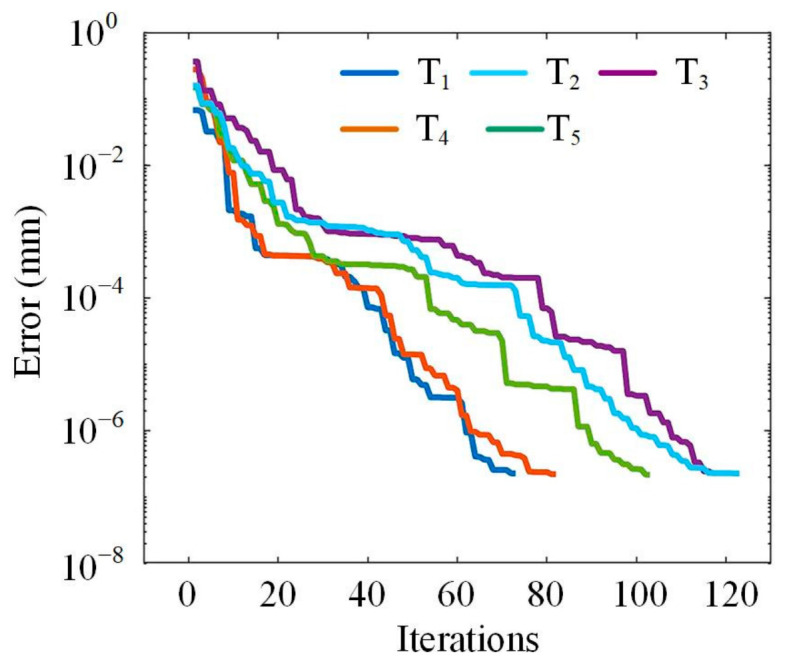
Convergence curves with different initial axes.

When the rotation axis has the aforementioned deviations, the average distance between the measurement points and the corresponding feature circles ranges from 0.08 mm to 0.40 mm. After applying the optimization method, the rotation axis converges to the ideal position. The average distance between rotated feature points and ideal circles can be reduced to 2.278 × 10^−7^ mm for all the cases, as shown in Figure 12. This convergence error can be negligible for practical measurements. It demonstrates that the proposed method achieves good convergence under varying initial positions, validating the rationality of the proposed method.

### 5.5. Experimental Verification of Axis Optimization

**Figure 13:**
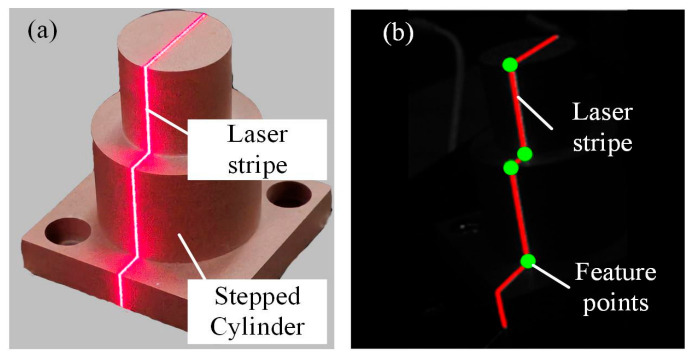
Feature points calculation with the stepped cylinder (**a**) The stepped cylinder (**b**) Feature point extraction process.

The stepped cylinder, as shown in Figure 13a, is machined on a high-speed machining center (JDGR200T, Beijing JingDiao Group, Beijing, China). This cylinder is then measured by the coordinate measuring machine (Global 7107, Hexagon, Qingdao, China). The diameter of small cylinder is 23.051 mm, with a height of 22.003 mm. The large cylindrical diameter is 36.006 mm, with a height of 25.005 mm. These measurement results are taken as reference values. The laser plane intersects the cylinder, generating a deformed stripe that consists of six-line segments. Pixel coordinates of the feature points are calculated using the method described in Section 4.2 and shown in Figure 13b. The turntable rotates with an incremental step of 4°. For each step, four feature points can be obtained. After a full rotation, a total of 360 feature points are acquired in the sensor coordinate system.

**Figure 14:**
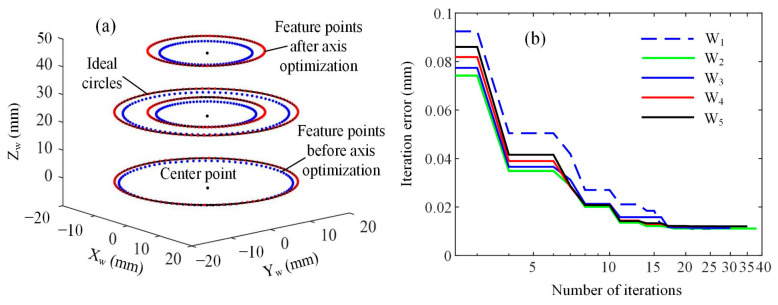
Optimization of the rotation axis. (**a**) Comparison of feature points before and after optimization, (**b**) convergence process with different initial rotation axes.

Using Equation (11), the feature points are rotated around the initial rotation axis to obtain the corresponding feature circle. By optimizing the coordinates of **P***_a_* and **P***_b_*, the average distance between feature points and ideal circles can be reduced, and a more accurate rotation axis can be obtained. The results before and after optimization are shown in Figure 14a. After optimization, the feature points align much better with the ideal circles, which demonstrates a substantial improvement in the rotation axis accuracy. As shown in Figure 14b, the average distance error declines continuously during optimization and finally converges to 0.011 mm.

The proposed axis optimization method is unaffected by errors generated in other calibration procedures, which enables remarkable accuracy improvement. To verify the reliability, the stepped cylinder was remounted on the turntable five times with different relative poses to the rotation axis, denoted by W_1_, W_2_, …, W_5_. The initial rotation axis was calculated using the same method discussed in Section 4.1. As shown in Figure 14b, significant differences can be observed for each installation. These initial values were obtained using the checkerboard target method. The variant initial values indicate that the initial rotation axis determined by the checkerboard is of low accuracy. While, by adopting the proposed optimization method, the average distances consistently converge to the range from 0.011 mm to 0.012 mm, with the variation of only 1 μm. The comparative results comprehensively prove that the proposed optimization method possesses outstanding reliability and high measurement accuracy.

## 6. Measurement Results and Analysis

### 6.1. Accuracy Evaluation of the Stepped Cylinder

To access the system accuracy, the stepped cylinder is measured using the rotation axes before and after optimization. The cylinder is re-mounted five times, with a positional deviation from the turntable axis introduced in each trial. Point clouds acquired from the large and small cylindrical sections are fitted to obtain the diameters. Reference diameters for the small and large cylinders are 23.051 mm and 36.006 mm, respectively. The corresponding diameter measurement errors are summarized in Table 2.

The average diameter error of the small cylinder before optimization is 0.3148 mm. It can be reduced to 0.0312 mm after optimization, a reduction of 90.09%. For the large cylinder, the average diameter error before optimization is 0.4204 mm, which decreases to 0.0266 mm after optimization, resulting in a reduction of 93.67%.

### 6.2. Accuracy Evaluation by Measuring of Typical Parts

**Figure 15:**
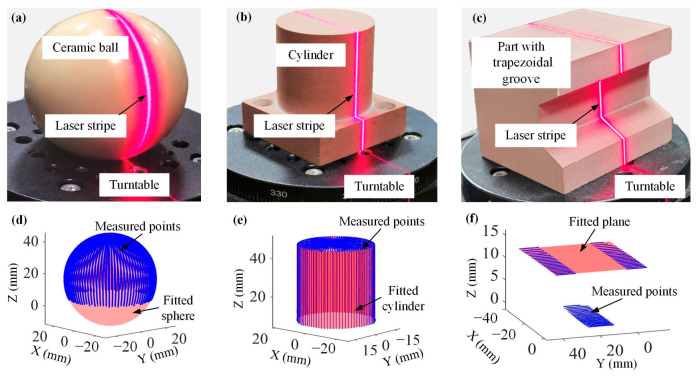
Accuracy evaluation by measuring different parts. (**a**) Ceramic ball, (**b**) Cylinder, (**c**) Part with trapezoidal groove, (**d**) point cloud and fitted results of the ball, (**e**) point cloud and fitted results of the cylinder, (**f**) measurement results of the trapezoidal groove.

To further verify the proposed method, the system is applied to the measurement of a ceramic ball, a cylinder, and a trapezoidal groove, as shown in Figure 15a–c. Figure 15d–f show the measured point clouds and the fitting results. For the ceramic ball, spherical fitting is performed. While the RANSAC method needs to be adopted for the cylindrical fitting due to the unwanted points on the top surface. For the trapezoidal groove, the top plane of the groove is fitted, and the depth of the groove is achieved by computing the distance between the points at the groove bottom and the fitted plane.

The reference values of these parts are obtained using the same coordinate measuring machine in Section 5.5. For the ceramic ball, the reference radius is 28.475 mm. Using the optimized rotation axis, 3D scanning points can be achieved. The fitting results from five repeated experiments are listed in Table 3, with an average error of 0.0059 mm and a maximum error of 0.0136 mm. For the cylinder part, its reference diameter is 38.004 mm. Five repeated experiments are performed, and the fitting results are shown in Table 4. The average error is 0.0221 mm, and the maximum error is 0.0279 mm. For the trapezoidal groove, the reference depth is 12.003 mm. The repeated measurement results are presented in Table 5, with a maximum error of 0.0175 mm, a minimum error of 0.0025 mm, and an average relative error of 0.0871%. Overall, the measured values are in close agreement with the reference values. For all samples, the maximum relative error is 0.1458%, which is less than 0.15%. This clearly demonstrates that the proposed method effectively improves the accuracy of rotary scanning system with LSLS.

### 6.3. Measurement of Complex Parts

**Figure 16:**
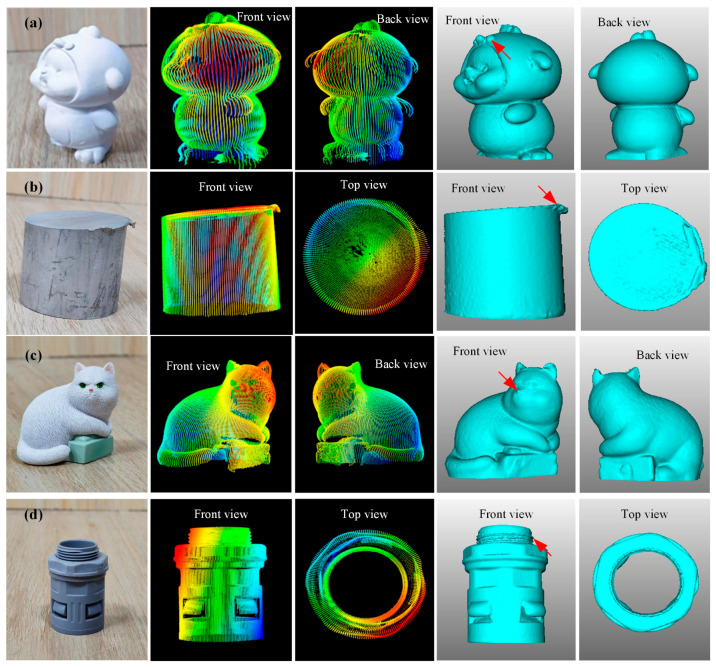
Measurement results of complex surfaces. (**a**) plaster model, (**b**) aluminum part with flash, (**c**) rubber model, (**d**) plastic pipe connector.

The system is also applied to measure complex surfaces, including a plaster model, an aluminum part with flash, a rubber model, and a plastic pipe connector. The Poisson algorithm is adopted for 3D surface reconstruction. The acquired point clouds and the reconstructed models are visualized from multiple viewpoints in Figure 16.

The reconstruction results demonstrate that the proposed line-structured light 3D measurement system can fully acquire 3D geometry of the parts. Surface details marked by the arrows, such as small protrusions, the flash on the aluminum part, the small spherical feature on the nose, and the threads on the plastic pipe connector, are clearly visible. These results further verify that the system integrated with a turntable enables accurate and full measurement of complex surfaces.

## 7. Conclusions

A rotation axis optimization method based on a stepped cylinder is proposed for line-structured light measurement system. The laser plane optimization is firstly proposed based on the principle of CRI. With the optimized laser plane, the RMSE of the sensor can be reduced from 0.021 mm to 0.003 mm. Using the stepped cylinder, precise feature points can be extracted from the cylinder edges. As the cylinder rotates, four sets of circularly distributed feature point sequences can be obtained. The optimal rotation axis is solved by minimizing the average distance between the point sequence and the corresponding ideal circles. With optimized rotation axis, the measured diameter error of the stepped cylinder can be reduced by over 90%. Measurements of ceramic spheres, cylindrical features, and trapezoidal grooves show a maximum relative error of less than 0.15%, which proves a remarkable improvement in measurement accuracy. The system also consistently achieves complete surface measurements for objects with varying materials and complex shapes, further validating the feasibility and reliability of the calibration method.

## Figures and Tables

**Table 1:** Deviations added to two points on the rotation axis (unit: mm).

No.	Δa_x_	Δa_y_	Δa_z_	Δb_x_	Δb_y_	Δb_z_
T_1_	−0.3	−0.2	−0.1	−0.3	−0.2	−0.1
T_2_	+0.3	+0.2	+0.1	+0.3	+0.2	+0.1
T_3_	−0.8	−0.2	+0.3	−0.3	+0.2	+0.3
T_4_	+0.6	+0.4	+0.2	+0.5	+0.3	+0.2
T_5_	−0.4	−0.7	−0.2	−0.3	−0.4	+0.5

**Table 2:** Diameter error of the stepped cylinder before and after axis optimization (unit: mm).

No.	Small Cylinder			Large Cylinder		
	Before	After	Reduced By	Before	After	Reduced By
1	0.3260	0.0322	90.12%	0.2334	0.0084	96.40%
2	0.2693	0.0314	88.34%	0.4420	0.0431	90.25%
3	0.4149	0.0480	88.43%	0.4498	0.0542	87.95%
4	0.2796	0.0197	92.95%	0.5203	0.0044	99.15%
5	0.2843	0.0248	91.28%	0.4565	0.0229	94.98%
Average	0.3148	0.0312	90.09%	0.4204	0.0266	93.67%

**Table 3:** Measurement results of the ceramic ball with different position shifts (unit: mm).

No.	1	2	3	4	5
Diameter	28.4734	28.4670	28.4716	28.4687	28.4723
Error	0.0015	0.0079	0.0033	0.0062	0.0026
Relative error	0.0052%	0.0277%	0.0116%	0.0218%	0.0091%

**Table 4:** Measurement results of the cylinder with different position shifts (unit: mm).

No.	1	2	3	4	5
Diameter	38.0278	37.9893	37.9761	38.0282	38.0218
Error	0.0258	0.0147	0.0279	0.0242	0.0178
Relative error	0.0679%	0.0387%	0.0734%	0.0637%	0.0469%

**Table 5:** Measurement results of trapezoidal groove with different position shifts (unit: mm).

No.	1	2	3	4	5
Height	12.0172	12.0204	12.0090	12.0151	12.0005
Error	0.0142	0.0175	0.0060	0.0121	0.0025
Relative error	0.1183%	0.1458%	0.0500%	0.1008%	0.0208%

## Data Availability

The original contributions presented in this study are included in the article. Further inquiries can be directed to the corresponding authors.

## References

[1] Liu B., Wu R., Liu Y. (2020). Calibration algorithm for error screening based on line structured light. Int. J. Artif. Intell. Tools.

[2] Javaid M., Haleem A., Singh R.P., Suman R. (2021). Industrial perspectives of 3D scanning: Features, roles and it’s analytical applications. Sens. Int..

[3] Chi S., Xie Z., Chen W. (2016). A laser line auto-scanning system for underwater 3D reconstruction. Sensors.

[4] Ren Z., Fang F., Yan N., Wu Y. (2022). State of the art in defect detection based on machine vision. Int. J. Precis. Eng. Manuf.-Green Technol..

[5] Cui B., Tao W., Zhao H. (2021). High-precision 3D reconstruction for small-to-medium-sized objects utilizing line-structured light scanning: A review. Remote Sens..

[6] Cheng A., Lu S., Gao F. (2023). Anomaly detection of tire tiny text: Mechanism and method. IEEE Trans. Autom. Sci. Eng..

[7] Li Y., Zhou J., Mao Q., Jin J., Huang F. (2020). Line structured light 3D sensing with synchronous color mapping. IEEE Sens. J..

[8] Chang H., Li D., Zhang X., Cui X., Fu Z., Chen X., Song Y. (2024). Real-time height measurement with a line-structured-light based imaging system. Sens. Actuat. A—Phys..

[9] Chen L., He J., Wu Y., Tang Y., Ge G., Wang W. (2024). Detection and 3D visualization of human tooth surface cracks using line structured light. IEEE Sens. J..

[10] Zhang Z. (2000). A flexible new technique for camera calibration. IEEE Trans. Pattern Anal. Mach. Intell..

[11] Qiu Z., Xiao J. (2019). New calibration method of line structured light vision system and application for vibration measurement and control. Opt. Precis. Eng..

[12] Zou W., Wei Z., Liu F. (2019). High-accuracy calibration of line-structured light vision sensors using a plane mirror. Opt. Express.

[13] Shao M., Dong J., Madessa A.H. (2019). A new calibration method for line-structured light vision sensors based on concentric circle feature. J. Eur. Opt. Soc.-Rapid Publ..

[14] Wang L., Zhou Q., Fang Y., Wang S., Li G. (2021). Detection method of rail fastener fastening state based on line structured light. Laser Optoelectron. Prog..

[15] Wang M., Sun Q., Gao C., Ren Z., Dai W. (2023). A three-dimensional vision measurement method based on double-line combined structured light. Sci. Rep..

[16] Ha V., Do V., Lee B. (2024). Calibration method of a three-dimensional scanner based on a line laser projector and a camera with 1-axis rotating mechanism. Opt. Eng..

[17] Zhang Z., Huang Q., Zhu L., Li P. (2024). A Surface Inspection Method for Rotary Workpieces Using Line Structured Light Based on 3D Point Cloud Reconstruction. https://ssrn.com/abstract=4725568.

[18] Chen P., Dai M., Chen K., Zhang Z. (2014). Rotation axis calibration of a turntable using constrained global optimization. Optik.

[19] Liu C., Fu X., Duan F., Li T., Li J., Wang R. (2023). A novel method to calibrate the rotation axis of a line-structured light 3-dimensional measurement system. Opt. Laser Eng..

[20] Li Z., Fang C., Zhang X. (2025). A full-profile measurement method for an inner wall with narrow-aperture and large-cavity parts based on line-structured light rotary scanning. Sensors.

[21] Hou Y., Su X., Chen W. (2020). Alignment method of an axis based on camera calibration in a rotating optical measurement system. Appl. Sci..

[22] Liu X., Wang Z.J., Yao P. (2022). Measurement and error compensation of 3D morphology with precision rotation line structured light. Chin. J. Lasers.

[23] Wang T., Chang Y., Yu Z., Zhang Z., Zhang Y., Liu J., Liu X., Li L. (2025). Single-view iterative measurement of rotary axis radial error motion utilizing line-structured light. Meas. Sci. Technol..

[24] Cai X., Zhong K., Fu Y., Chen J., Liu Y., Huang C. (2020). Calibration method for the rotating axis in panoramic 3D shape measurement based on a turntable. Meas. Sci. Technol..

[25] Niu Z., Liu K., Wang Y., Huang S., Deng X., Zhang Z. (2017). Calibration method for the relative orientation between the rotation axis and a camera using constrained global optimization. Meas. Sci. Technol..

[26] Zong Y., Liang J., Pai W., Ye M., Ren M., Zhao J., Tang Z., Zhang J. (2022). A high-efficiency and high-precision automatic 3D scanning system for industrial parts based on a scanning path planning algorithm. Opt. Laser Eng..

[27] Ye Y., Song Z. An accurate 3D point cloud registration approach for the turntable-based 3D scanning system. Proceedings of the 2015 IEEE International Conference on Information and Automation.

[28] Zhu K., Gong L., Gu D., Liu C. An analytic calibration method for turntable-based 3D scanning system. Proceedings of the 2019 IEEE/ASME International Conference on Advanced Intelligent Mechatronics (AIM).

[29] Zuo C., Qian J., Feng S., Yin W., Li Y., Fan P., Han J., Qian K., Chen Q. (2022). Deep learning in optical metrology: A review. Light Sci. Appl..

[30] Zhang Z., Kong L., Zhang L., Pan X., Das T., Wang B., Liu B., Wang F., Nape I., Shen Y. (2025). Structured light meets machine intelligence. eLight.

[31] Cao H., Qiao D., Han M., Yu W., Wang B., Shen Y. (2026). Geometry-aware super-resolution fusion calibration for binocular structured light 3D reconstruction. Commun. Phys..

[32] Guo X., Shi Z., Yu B., Zhao B., Li K., Sun Y. (2020). 3D measurement of gears based on a line structured light sensor. Precis. Eng..

[33] Wei P., Yang W. (2025). An SQP-type proximal gradient method for composite optimization problems with equality constraints. J. Comput. Math..

[34] Li Y., Zhou J., Huang F., Liu L. (2017). Sub-pixel extraction of laser stripe center using an improved gray-gravity method. Sensors.

[35] Fischler M.A., Bolles R.C. (1981). Random sample consensus: A paradigm for model fitting with applications to image analysis and automated cartography. Commun. ACM.

[36] Tsai R. (1987). A versatile camera calibration technique for high-accuracy 3D machine vision metrology using off-the-shelf TV cameras and lenses. IEEE J. Robot. Autom..

